# DKPE-GraphSYN: a drug synergy prediction model based on joint dual kernel density estimation and positional encoding for graph representation

**DOI:** 10.3389/fgene.2024.1401544

**Published:** 2024-06-14

**Authors:** Yunyun Dong, Yujie Bai, Haitao Liu, Ziting Yang, Yunqing Chang, Jianguang Li, Qixuan Han, Xiufang Feng, Xiaole Fan, Xiaoqiang Ren

**Affiliations:** ^1^ School of Software, Taiyuan University of Technology, Taiyuan, Shanxi, China; ^2^ Information Management Department, Shanxi Provincial People’s Hospital, Taiyuan, Shanxi, China

**Keywords:** drug-drug interaction prediction, drug combination, synergistic effect, cancer treatment, graph attention network, deep learning

## Abstract

**Introduction:** Synergistic medication, a crucial therapeutic strategy in cancer treatment, involves combining multiple drugs to enhance therapeutic effectiveness and mitigate side effects. Current research predominantly employs deep learning models for extracting features from cell line and cancer drug structure data. However, these methods often overlook the intricate nonlinear relationships within the data, neglecting the distribution characteristics and weighted probability densities of gene expression data in multi-dimensional space. It also fails to fully exploit the structural information of cancer drugs and the potential interactions between drug molecules.

**Methods:** To overcome these challenges, we introduce an innovative end-to-end learning model specifically tailored for cancer drugs, named Dual Kernel Density and Positional Encoding (DKPE) for Graph Synergy Representation Network (DKPEGraphSYN). This model is engineered to refine the prediction of drug combination synergy effects in cancer. DKPE-GraphSYN utilizes Dual Kernel Density Estimation and Positional Encoding techniques to effectively capture the weighted probability density and spatial distribution information of gene expression, while exploring the interactions and potential relationships between cancer drug molecules via a graph neural network.

**Results:** Experimental results show that our prediction model achieves significant performance enhancements in forecasting drug synergy effects on a comprehensive cancer drug and cell line synergy dataset, achieving an AUPR of 0.969 and an AUC of 0.976.

**Discussion:** These results confirm our model’s superior accuracy in predicting cancer drug combinations, providing a supportive method for clinical medication strategy in cancer.

## 1 Introduction

Predicting drug combinations is a pivotal task in contemporary medication strategies, focusing on optimizing therapeutic outcomes by anticipating the interactions between different drugs and addressing the limitations of single drugs through predicting interactions between different drugs (Mokhtari et al., 2017). Research on drug combination prediction is of great importance for achieving personalized medicine and enhancing treatment effectiveness (Csermely et al., 2013). With the rapid development of deep learning technologies, the field of drug combination prediction is experiencing significant technological innovations, greatly advancing research in this area (Güvenç Paltun et al., 2021). In 2018, Preuer et al. (2018) developed the DeepSynergy model, marking a significant milestone in the application of deep learning methods for predicting drug combinations. Inspired by DeepSynergy, researchers developed the DeepSignalingSynergy model in 2020 (Zhang et al., 2020), based on a sparse network construction. This model uses neurons in the hidden layer to simulate signaling pathways in cancer cells, assessing each neuron’s contribution to the final prediction through hierarchical relevance propagation (Montavon et al., 2019), thereby enhancing the model’s interpretability. While the DeepSynergy model introduces a novel approach to forecasting drug interactions, it faces limitations when dealing with intricate gene expression profiles and the varied molecular architectures of drugs. Kuru et al. (2021) proposed the MatchMaker model, training a subnetwork for each drug-cell combination. The output latent representations of these subnetworks are concatenated to serve as inputs for another subnetwork, used for predicting synergy scores. Building on this, MARSY (El Khili et al., 2023) also trained two subnetworks representing drug pairs and drug-drug-cell triple combinations; Additionally, CCSynergy (Hosseini and Zhou, 2023) and SynPathy (Tang and Gottlieb, 2022) are drug combination synergy prediction models developed in recent years based on the DeepSynergy model, integrating more types of drug and cell characteristics.

Despite significant progress with deep learning-based drug combination prediction models like DeepSynergy, substantial challenges remain in handling complex gene expression data and drug structural diversity. Subsequent research, such as DrugCell (Kuenzi et al., 2020), has employed more complex network architectures and data processing methods to address these challenges. These methods include advanced dimensionality reduction techniques and modeling for specific biological pathways, aiming to improve the predictive accuracy and biological interpretability. The development and application of Graph Neural Networks (GNNs) (Scarselli et al., 2008) have provided new methods for representing molecular structures and cellular network characterizations. Recent models such as GraphSynergy (Yang et al., 2021), PRODeepSyn (Wang et al., 2022), MOOMIN (Rozemberczki et al., 2022), and KGE-DC (Zhang and Tu, 2022) apply GNNs to drug combination synergy prediction, offering new methods to better capture the intricate interactions and relationships within the data.

While deep learning approaches have achieved some progress in predicting drug combination synergy, they exhibit limitations, particularly in adequately addressing the spatial distribution characteristics of gene expression data. This oversight leads to an in-complete understanding of the spatial adjacency relationships among genes. Furthermore, the acquisition of structured regularity information concerning drugs, as well as potential associative features between drug molecules, remains insufficient.

To overcome the limitations of current methodologies, we introduce a novel predictive model named DKPE-GraphSYN. The main contributions are as follows:

• **DKPE-GraphSYN model:** The proposed DKPE-GraphSYN model employs a Dual Kernel Density Estimation (DKDE) and Positional Encoding (PE) Channel Cascade Algorithm for processing gene expression data. The innovative method encapsulates both locational and weighted probability density information, thereby effectively capturing the spatial distribution and weights of genes within cellular responses.
• **Graph Representation of Drug Molecules:** By representing drug molecules using a graph structure, our model captures the potential associations between drug molecules, enhancing our understanding of their interactions.
• **Integration of Convolutional Neural Networks (CNN) and GNN:** The DKPE-GraphSYN model integrates CNN with GNN to effectively capture associative features between cell lines and drugs, thus enhancing the accuracy of predicting drug combination effects.

## 2 Materials and methods

Our research method commenced with dividing the dataset evenly into five parts, proceeding through five phases of training and verification. In each phase, we designated one part as the validation set and used using the rest for training set. This rotation ensures all data serve in validation and training sets. We assess the model’s validation performance in each phase, and the collective findings from all phases culminate in a comprehensive evaluation of the model’s effectiveness.

### 2.1 Datesets

Gene expression data for human cancer cell lines were obtained from the GDSC (Genomics of Drug Sensitivity in Cancer) database (Yang et al., 2012), a comprehensive repository encompassing cancer genomics and drug sensitivity. We selected data for approximately 1000 cancer cell lines from the GDSC. For each cell line, normalized expression levels of 17,737 genes were recorded, from which we selectively identified 496 landmark genes for our analysis. Additionally, the database converted protein/RNA markers derived from gene expression into numerical sequences, facilitating their analysis through computational methods. Subsequently, these gene expression vectors were normalized to standardize the data range, facilitating further analysis.

Our dataset of drug combinations was gathered from the DrugComb database (Zagidullin et al., 2019) (https://drugcomb.org/), derived from 34 diverse studies including O'NEIL (Merck) (O’Neil et al., 2016), CLOUD and so on. Initially comprising 1,432,351 samples, we selected triples consisting of drug A, drug B, and a cell line. We excluded any samples that lacked complete information on drug names, cell lines, or synergy scores to ensure the dataset contained only complete information for analysis. Additionally, to avoid data redundancy, we identified and removed duplicate triples.

By integrating the GDSC and DrugComb databases at the cellular line level, we have successfully established a balanced benchmark dataset consisting of 25,758 unique drug-drug-cell line combinations.

### 2.2 Drug synergy reference scores

In this study, we define positive (synergistic) and negative (antagonistic) samples based on four types of synergy scores: Loewe (Loewe, 1953), Bliss (Bliss, 1939), HSA (Me, 1989), and ZIP (Yadav et al., 2015). Synergy scores within the DrugComb database are computed utilizing the SynergyFinder software (Ianevski et al., 2017). Relying on a single type of synergy score for evaluation can introduce inaccuracies. Therefore, we utilize a variety of threshold scores (Loewe, Bliss, HSA, ZIP) for assessment purposes. A sample is only incorporated into the training set when all four scores unanimously classify a drug combination as either a positive or a negative sample. This methodology is designed to provide a more detailed and accurate assessment of drug synergy effects, thereby enhancing the precision and stability of the predictive model.

### 2.3 Architecture

**FIGURE 1:**
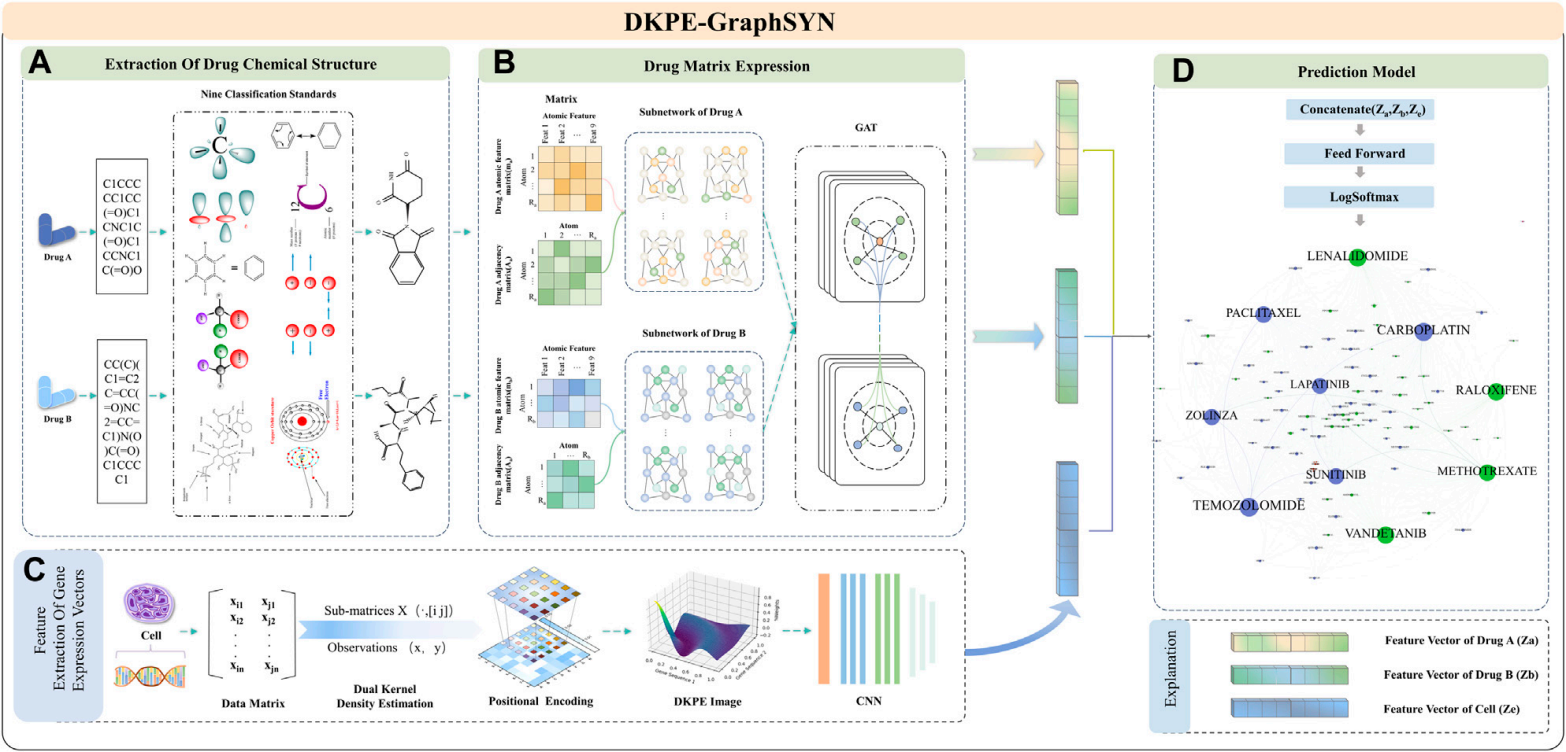
Feature Extraction and Model Training Process of DKPE-GraphSYN. Part **(A)** The chemical structure of drugs A and B is extracted based on their SMILES representation and nine chemical property classification standards. Part **(B)** The molecular properties and interactions of the drugs are represented through a graph structure network using matrices. Part **(C)** Features of the gene expression vector of the cell line are extracted using a DKDE and PE channel cascade algorithm. Part **(D)** A classification model is constructed by merging the drug and gene expression vector feature vectors, utilizing a feedforward network and the LogSoftmax function to predict the synergistic effects of drugs.

As shown in Figure 1, DKPE-GraphSYN is composed of the DKDE and PE joint gene expression vector feature extraction module, and a graph structure characterization module for drug molecular structure features. In the dual kernel density gene expression vector module, we introduce a DKDE and PE channel cascade algorithm for the first time (see Algorithm 1 for details). This algorithm captures the weighted density and spatial distribution features of gene expression vector data. By cascading DKDE and PE channels, we generate a DKPE image of the gene expression vector, incorporating both DKDE and PE features, which serves as the input for the CNN.

In the graph structure characterization module for drug molecular features, atoms and bonds in the chemical structure of drugs are considered as nodes and edges of the graph (Reiser et al., 2022), respectively. The regularity features of the drug graph structure are then extracted using a GNN. Finally, our model integrates the embedding results from the CNN and GNN, processes them through a feedforward neural network, and outputs the predicted interaction scores between drugs to categorize synergistic and antagonistic interactions.

#### 2.3.1 Feature extraction of gene expression vectors

We propose a DKDE and gene PE for channel cascade integration to comprehensively analyze gene expression vector data, considering not only the weighted probability density of genes but also their spatial distribution. DKDE enhances the understanding of gene pair distributions in gene expression data by integrating calculations from Gaussian kernel functions with estimations of probability distributions, thereby uncovering the weighted probability distribution of gene pairs, and reflecting their interaction strength and spatial distribution characteristics, PE assigns spatial coordinates as numerical codes to each grid point in the DKDE map, to reflect the relative positional relationships and proximal associations of gene expression data in spatial distribution. The channel cascade integration method combines the weighted probability density information of gene expression obtained from DKDE with spatial PE into an enhanced feature matrix. This facilitates convolutional neural networks in concurrently processing and evaluating the spatial distribution characteristics and probability density of genes.

#### 2.3.2 Dual kernel density estimation

In the preprocessing stage of dual kernel probability estimation, representative gene expression vectors are first extracted from the gene expression dataset, with each cell line’s feature vector having a length of 496. Subsequently, each vector is evenly divided into two parts, which are considered as two variables i and j in the data matrix X . A probability distribution matrix is then obtained through the DKDE algorithm. In this matrix, each point represents a weighted probability value of gene expression, indicating a certain probability density, that reflect the interaction strength of specific gene expression pairs. By calculating all points on an equidistant grid, we create a DKDE map, which details the interaction strength of gene expression pairs.

**FIGURE 2:**
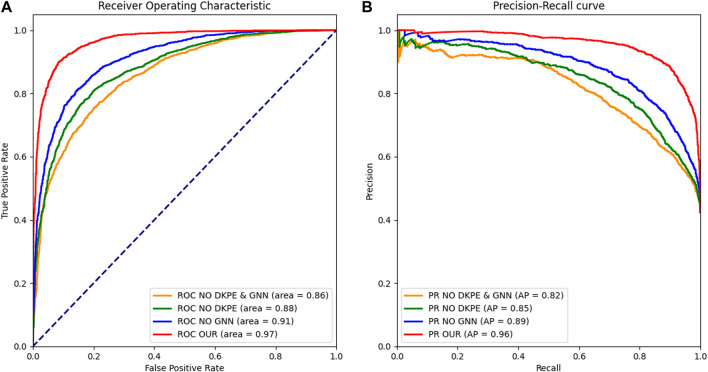
Receiver Operating Characteristic and Precision Recall Curve Comparison. **(A)** The left panel displays the ROC curves for different model variants, with the AUC metric indicating the ability to distinguish between synergistic and non-synergistic drug combinations. **(B)** The right panel shows the PR, with the AUPR metric reflecting the precision and recall balance of the models in predicting drug synergies. The ROC and PR curves are essential for evaluating the performance of the ablation study models, demonstrating the impact of the DKPE and GNN components on predictive accuracy.

The DKDE algorithm utilizes the Kernel Density Estimation (KDE) function to compute weighted probability densities. This KDE function, a non-parametric statistical approach, is applied to approximate unknown density functions. As illustrated in Figure 2, the algorithm initially extracts columns corresponding to variables i and j from the gene data matrix X , forming an n×2 submatrix $X\left(\cdot ,\left[ij\right]\right)$ . For every observed value pair $\left(x,y\right)$ within the submatrix $X\left(\cdot ,\left[ij\right]\right)$ , which denotes gene expression, the value of kernel function *K* is determined, with the Gaussian kernel selected as *K*. The formula for calculating the kernel function is shown in Eq. 1:

$$K\left(u,v\right)=\left(\frac{1}{2\pi }\right)*\exp\left(-0.5*\left(u^{2}+v^{2}\right)\right),$$ (1)

where $u=\frac{x-x_{i}}{h},v=\frac{y-y_{j}}{h}$ , with $x_{i}$ and $y_{j}$ being the observed values (i.e., gene samples), and h as the bandwidth. The sum of all kernel function K values is then divided by the product of the total count of observations n and the bandwidth $h^{2}$ , to calculate the value of the bivariate kernel density estimation $f_{ij}$ . The calculation process is shown in Eq. 2.

$$f_{ij}\left(x,y\right)=\left(\frac{1}{n*h^{2}}\right)*\sum K\left(\frac{x-x_{i}}{h},\frac{y-y_{j}}{h}\right).$$ (2)

By repeating the above steps and for all points on an equidistant grid, we obtain the values of the bivariate kernel density estimation $f_{ij}\left(x,y\right)$ , which form the DKDE map. This map reveals the interaction strength of gene expression pairs, where $f_{ij}\neq f_{ji}$ indicates the asymmetry of the estimated values, further emphasizing the directional characteristics of interaction strength between gene pairs.

#### 2.3.3 Grid position encoding

Grid Position Encoding forms the core component of our DKDE and PE Channel Cascade Algorithm, designed to precisely capture and describe the complex distribution and interrelations of gene expression data in multidimensional space. By assigning a unique positional encoding to each data point in the DKDE map, we can not only quantitatively analyze the interaction strength of gene pairs but also reveal their spatial relevance in the cellular functional structure.

The procedural steps for applying the PE technique start with identifying the two-dimensional coordinates $\left(a,b\right)$ for each gene expression data point on the DKDE map, representing its actual physical location in the kernel density estimation map. To integrate these spatial coordinates into the model with the numerical range of other model features, the coordinates are normalized and align them to values within the $\left[0,1\right]$ range. The formula for normalized PE is as follows:

$$\left(a,b\right)=\left(\frac{a-a_{\min}}{a_{\max}-a_{\min}},\frac{b-b_{\min}}{b_{\max}-b_{\min}}\right),$$ (3)

where $a_{\min}$ and $b_{\min}$ , respectively represent the minimum values of all coordinates in the DKDE map, while $a_{\max}$ and $b_{\max}$ represent the maximum values of all coordinates. This normalization process converts the interaction strength of each gene pair and their relative position within the cell into normalized feature vectors, thereby providing comprehensive information for model analysis. This method is particularly effective for analyzing spatial interactions between genes, capable of identifying certain genes that may coparticipate in the same biological processes or signaling pathways due to their proximal locations within the cell.

Algorithm 1 DKDE and PE Channel Concatenation Algorithm.

Input: Gene expression dataset, with each cell line’s feature vector length being 496
Output: DKPE image indicating the interaction strength and PE of gene expression pairs
Step 1: Preprocessing
For each cell line in dataset X

Extract representative gene expression vectors from the gene expression dataset
Derive characteristic gene expression vectors from the gene expression dataset
Evenly split each vector into two parts, defined as variables i and j

Step 2: DKDE
Initialize an empty probability distribution matrix P

For each pair of variables $\left(i,j\right)$

 Extract columns corresponding to variables i and j from data matrix, forming submatrix $X\left(\cdot ,\left[ij\right]\right)$

 For each pair of observed values $\left(x,y\right)$ in submatrix $X\left(\cdot ,\left[ij\right]\right)$

  Calculate kernel function $K\left(u,v\right)$ *,* where $u=\frac{x-x_{i}}{h},v=\frac{y-y_{j}}{h}$

  Calculate and sum kernel function values to obtain $f_{ij}\left(x,y\right)$

 Store $f_{ij}\left(x,y\right)$ in the corresponding position of probability distribution matrix P

Step 3: PE
For each point in probability distribution matrix P

Assign its position coordinates in the DK graph as PE, and perform normalization: $\left(a,b\right)=\left(\frac{a-a_{\min}}{a_{\max}-a_{\min}},\frac{b-b_{\min}}{b_{\max}-b_{\min}}\right)$

Step 4: Channel Concatenation
For each gene expression pair $\left(i,j\right)$

 Create a feature matrix F containing kernel density estimation values $f_{ij}\left(x,y\right)$ and PE
 Treat kernel density estimation values and PE as separate data channels.
Concatenate these data channels to form an enhanced feature matrix F , where each element contains the kernel density estimation value $f_{ij}\left(x,y\right)$ and the PE of the corresponding grid point. Each feature vector contains both kernel density estimation values and positional information
Step 5: Generating DKPE image
Use the matplotlib plotting library to transform the enhanced feature matrix F into a DKPE image
Utilize the results from Steps 2- Step 5 to generate a DKPE image for each pair of gene expression variables i and j

These DKPE image reveal the interaction strength and spatial distribution information of gene expression pairs
Step 6: Output
Output the DKPE image for all gene expression pairs
End

#### 2.3.4 Channel cascading

Channel cascading constitutes a key component of our DKDE and PE algorithm, aiming to effectively integrate the DKDE values and spatial location information of gene expression data. Initially, the algorithm generates a DKDE map through dual kernel density estimation, focusing on each gene expression variable pair i and, to precisely delineate the interaction intensity between gene expression pairs. During the creation of the DKDE map, each gene expression point undergoes normalized coordinate encoding, converting the position information of each point into standardized values. This normalization process follows Formula 3. In the channel cascading stage, the kernel density estimation values $f_{ij}\left(x,y\right)$ for each pair of gene expressions and the normalized positional encoding are considered as independent data channels. Subsequently, through a cascading merging strategy, these two data channels are combined to create a more enriched data layer. The merging operation is conducted on the channel dimension, aligning the kernel density estimation values and PE data side by side, facilitating a multidimensional integration of data.

The final enhanced feature matrix integrates the KDE values from the DKDE map with the normalized PE of each grid point. This improved feature matrix serves as the input to the CNN, aiming to intricately map out the complex spatial distribution characteristics of gene expression data. This innovative approach, enhances the data representation capability and provided richer information for predictive analysis, thereby significantly improving the model’s performance in gene expression data analysis.

#### 2.3.5 DKPE image feature expression network

The gene expression DKPE maps generated based on the a forementioned algorithm will serve as inputs for the DKPE Image Feature Expression Network. This architecture, based on a CNN framework, is specifically designed for processing and analyzing DKPE Images that integrate DKDE and the corresponding spatial positional encoding. The DKPE Image Feature Expression Network utilizes a ResNet-54 architecture (He et al., 2016), comprising a backbone network and five stage wise ResNet modules, each stage consisting of 3, 4, 6, 3, and 3 residual units respectively, and each stage is equipped with multiple fully connected layers to enhance the recognition and learning capabilities for complex gene expression patterns. Within the ResNet-54 architecture, the first ResNet module of each stage is responsible for feature map down-sampling in terms of spatial dimensions, gradually enhancing the network’s feature extraction and recognition capabilities.

To enhance computational efficiency and optimize performance, 1 × 1 convolution kernels were implemented at both the input and output phases of each feature extraction layer within the CNN, facilitating channel down-sampling and up-sampling. This approach significantly lowers computational demands while maintaining the spatial resolution of the feature maps. Additionally, to mitigate the problem of neuron inactivity that can arise from using the conventional ReLU activation function, the Parametric Rectified Linear Unit (PReLU) was adopted as the activation function. The introduction of this function allows the model to adaptively learn the parameters of the negative slope, thereby enhancing both the model’s adaptability and robustness (Xie et al., 2017).

#### 2.3.6 Feature extraction of drug chemical structure

**FIGURE 3:**
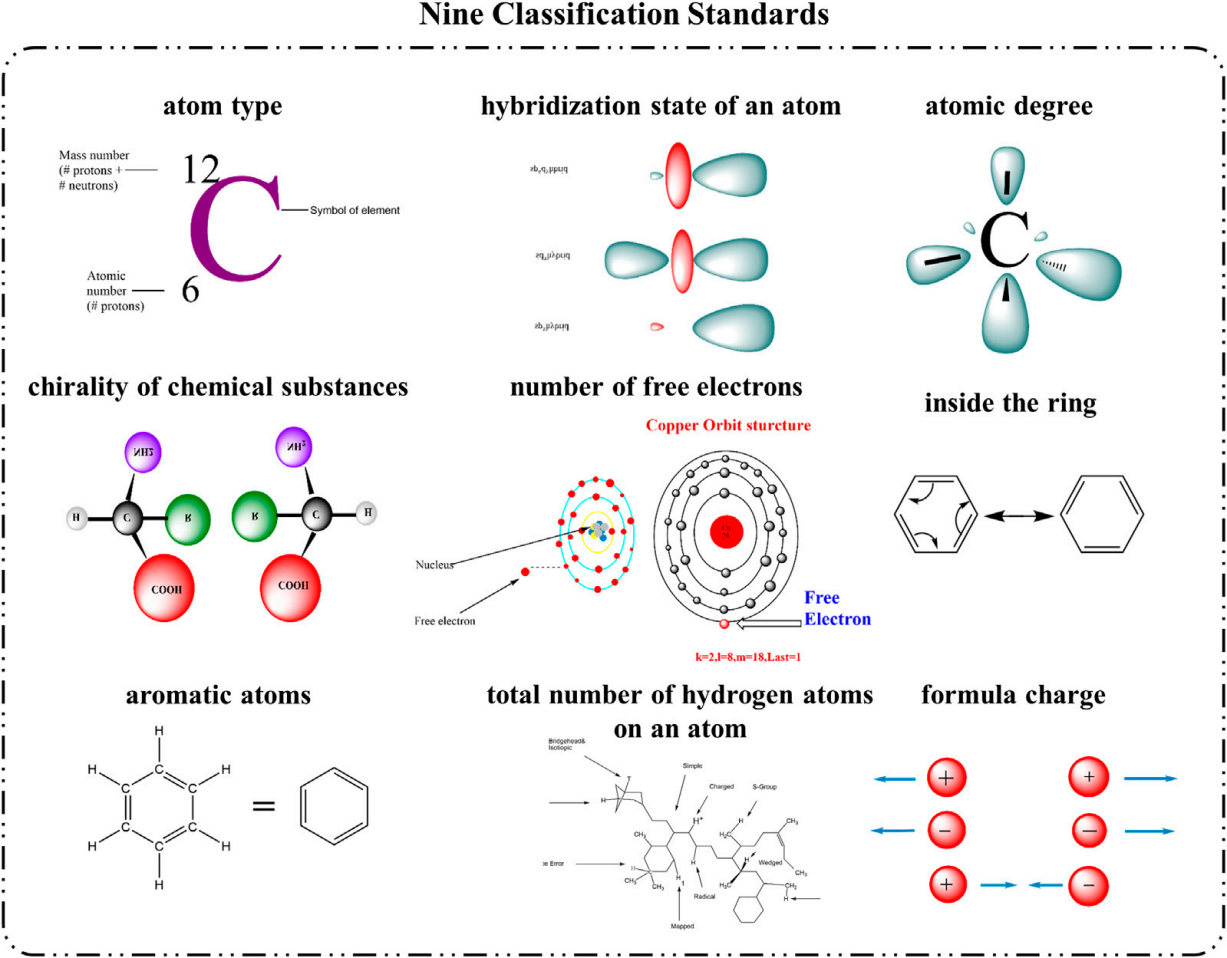
Nine Classification Standards for Drug Atom Characterization. This figure presents the nine key attributes used to classify the atoms within a drug’s chemical structure.

To capture the graph structural features of drug molecules and embed node representations, we designed a module for extracting graphical representations of drug molecules. This module utilizes GNN to extract embedded vectors that include information about the nodes and their neighborhoods, thereby learning the structural features of drug molecules and revealing the interrelationships between drugs. In processing the chemical structures of drugs from the DrugComb database, we adopted the SMILES notation and used the RDKit (Landrum, 2013) cheminformatics software package to extract features of each atom, as depicted in Figure 3. These features include encompasses atomic number, chirality, atom degree (counting hydrogen atoms), formal charge, total hydrogen atom count, number of free electrons, hybridization state, aromaticity, and whether the atom is part of a ring. Considering these nine types of atomic structural features, for the R atoms contained in each drug’s chemical structure, we constructed a feature matrix of dimensions R×9 , where R is the number of atoms (nodes) in the chemical structure graph. Moreover, for each drug, a binary adjacency matrix A with dimensions R×R was developed to represent the chemical compounds’ structural details. In this matrix, if a chemical bond exists between two atoms, the respective element in the matrix is assigned a value of 1; if there is no bond, it is set to 0. For drugs with a chemical structure containing R atoms, a feature matrix M of dimensions R×9 was constructed as shown in Eq. 4:

$$M={\left[m_{ij}\right]}_{\left(R\times 9\right)},$$ (4)

where $m_{ij}$ is the j−th feature value of the i−th atom.

A binary adjacency matrix A of dimensions R×R was constructed:

$$A={\left[a_{ij}\right]}_{\left(R\times R\right)},$$ (5)

where $a_{ij}=1$ indicates that a bond is formed between the i−th and j−th atoms, otherwise $a_{ij}=0$ .

#### 2.3.7 Graph neural network model

Considering the chemical composition of drugs, we establish a drug graph $G=\left(V,E\right)$ where the drug atoms depicted as nodes $V=\left\{1,\ldots ,A\right\}$ , and the connections between these atoms illustrated as edges E⊆V×V . Similar to feedforward networks, GNN consists of multiple layers, where L denotes the “depth” of the network. Particularly, every layer $l\in \left\{1,\ldots ,L\right\}$ signifies the l−hop neighborhood of a node (atom), representing the subgraph comprising all atoms accessible within l steps.

Node Representation: Each node (atom) i∈V is initially represented by a vector $h_{i}^{\left(0\right)}\in R^{9}$ . The neighbors of node i are defined as $N_{i}=\left\{j\in V|\left(j,i\right)\in E\right\}$ , which includes all nodes j that are connected to node i .The representation of node i at layer $\left(l+1\right)$ , denoted as $h_{i}^{\left(l+1\right)}$ , is obtained by aggregating the representations of its neighboring nodes. Therefore, each node’s representation is updated based on its neighbors’ representations at each layer. The update process is shown in Eq. 6.

$$h_{i}^{\left(l+1\right)}=\phi \left(\xi \left(\left\{h_{j}^{\left(l\right)}|j\in N_{i}\right\}\right)\right),$$ (6)

where ϕ and ξ are differentiable functions, and ξ is permutation invariant (i.e., typically invariant to order).

We utilize the Graph Attention Network version 2 (GATv2) operator (Brody et al., 2021) to update each $h_{i}$ (see Eq. 7). Compared to the traditional Graph Attention Network (GAT) (Velickovic et al., 2017), GATv2 exhibits two major advantages:

• The aggregation ξ of neighbor $j\in N_{i}$ is based on a weighted average of learned weights using an attention mechanism, rather than treating all neighbors as equally important.
• Each node can dynamically focus on any other node, implementing a flexible attention mechanism, in contrast to the static attention mechanism of the original GAT.

$$h_{i}^{\left(l+1\right)}=\phi \left(\alpha_{i,i}\Theta h_{i}^{\left(l\right)}+\sum_{j\in N_{i}}\;\alpha_{i,j}\Theta h_{j}^{\left(l\right)}\right).$$ (7)

The attention coefficient $\alpha_{i,j}$ is determined through a distinct process designed to gauge the relationship between two feature vectors. Within the attention framework, this coefficient is derived from the input feature vectors to identify the focal point. Various techniques can be employed to compute the attention coefficient, with the dot product or inner product frequently used to assess the similarity between two feature vectors. By computing and subsequently normalizing the dot product or inner product of feature vectors, a normalized attention weight is achieved.

By calculating the attention coefficients, we can determine which feature vectors or information are more important in the given input, thereby determining the allocation of attention or resources for the model’s subsequent processing steps. The attention coefficient $\alpha_{i,j}$ is obtained through a specific calculation method aimed at quantifying the correlation between two feature vectors. This process involves examining the input feature vectors to identify the focal point of attention. A frequently utilized technique for computing attention coefficients employs dot products or inner products for evaluating the resemblance among feature vectors. By normalizing these dot or inner products, we produce a uniform weight that signifies the significance of each feature vector. Therefore, the calculation of attention coefficients allows us to pinpoint key feature vectors or information in the input, and accordingly allocate attention and resources appropriately in further processing of the model. The attention coefficient $\alpha_{i,j}$ is calculated as shown in Eq. 8:

$$\alpha_{i,j}=\frac{\exp\left(a^{⊺}\text{LeakyReLU}\left(\Theta \left[h_{i}^{\left(l\right)}∥h_{j}^{\left(l\right)}\right]\right)\right)}{\sum_{k\in N_{i}\cup \left\{i\right\}}\;\exp\left(a^{⊺}\text{LeakyReLU}\left(\Theta \left[h_{i}^{\left(l\right)}∥h_{k}^{\left(l\right)}\right]\right)\right)},$$ (8)

a and Θ represent trainable parameters, while ϕ is the ELU (Exponential Linear Unit) activation function. Unlike the Rectified Linear Unit (ReLU), the ELU activation function addresses the limitation of ReLU producing zero for negative inputs. ELU is defined as shown in Eq. 9:

$$\phi \left(x\right)=\left\{\begin{array}{l} a*\left(\exp\left(x\right)-1\right),\;x<0\\ x,\;x\geq 0 \end{array}\right.$$ (9)

where a is a constant, typically positive. The ELU function exhibits nonlinear characteristics when x<0 and allows for negative outputs, thereby showing higher robustness compared to ReLU. In neural networks, the learned parameters a and ϕ are optimized through training data, with the aim of enhancing the network’s adaptability to training samples. The ELU activation function can be applied in both hidden and output layers, providing the network with greater representational power and activation range.

Graph representation method: This involves aggregating node representations to generate graph representations at a specific level. Specifically, by aggregating the updated node representations $h_{i}^{\left(l\right)}$ (using global average pooling), a graph representation $g^{\left(l\right)}$ at level l is generated:

$$g^{\left(l\right)}=\frac{1}{A}\sum_{n=1}^{A}\;h_{i}^{\left(l\right)}.$$ (10)

The GNN model utilizes learnable parameters of the global context vector c to calculate the attention weights for each pair of graph representations. The weighted average z of the graph representations $\left\{g^{\left(0\right)},\ldots ,g^{\left(L\right)}\right\}$ is ultimately calculated. This approach enables the model to consider global information while calculating graph representations, thereby generating more comprehensive and information rich graph representations.

$$\psi^{l}=\frac{\exp\left(score\left(c,g^{l}\right)\right)}{\sum_{j=1}^{L}\;\exp\left(score\left(c,g^{j}\right)\right)},$$ (11)

$$score\left(c,g^{l}\right)=\frac{c^{⊺}g^{l}}{\sqrt{d^{\prime }}},$$ (12)

$$z=\sum_{l=1}^{L}\;\psi^{l}g^{l}.$$ (13)

Among them, $g^{\left(l\right)}$ : the graph representation at layer l , which is the global average of all updated node representations $h_{i}^{\left(l\right)}$ ; A : the number of nodes in the graph; $h_{i}^{\left(l\right)}$ : the representation of the i−th node in the l−th layer; “ L ”: the total number of layers in the graph neural network, which is a hyperparameter; $\psi^{l}$ :the attention weights for the graph representation at layer l ; c : the global context vector, with learnable parameters; $\text{score}\left(c,g^{l}\right)$ : the similarity score between the global context vector c and the graph representation $g^{\left(l\right)}$ at layer l ; $d^{\prime }$ : the embedding dimension of the graph representation $g^{\left(l\right)}$ ; z : the final weighted average graph representation, obtained through the calculation of graph representations and corresponding attention weights across all layers.

Eq. 10 utilizes the global average pooling method to integrate all node representations at layer l, generating the graph representation $g^{\left(l\right)}$ for that layer. Eq. 11 calculates the attention weights $\psi^{l}$ for each layer’s graph representation using the softmax function, ensuring the sum of attention weights across all layers equal. Eq. 12 defines a scoring function to compute the similarity between the global context vector and each layer’s graph representation, using a normalized dot product method. Finally, Eq. 13 determines the final graph representation z by calculating the weighted sum of all layers’ graph representations and their corresponding attention weights. Within the input sample pairs, independent representation vectors for drugs, denoted as $z_{a}$ *and* $z_{b}$ , are determined for drug A and drug B, respectively.

#### 2.3.8 Classifier model

In our classification prediction module, the gene feature vector $z_{e}$ obtained from the dual kernel density gene expression module is first integrated with the feature vectors $z_{a}$ and $z_{b}$ of drugs A and B, respectively, processed by the graph structure representation module. The combined feature vector is then input into a feedforward neural network (FFNN) with two hidden layers. The FFNN employs the ReLU activation function and the dropout method for regularization. The output layer generates a two element vectors, which, after being processed by the LogSoftmax function, is used to output the predicted drug interaction score for the classification of synergistic and antagonistic effects.

## 3 Results

### 3.1 Experiment

In our study, we employed a stringent 5-fold cross validation approach to assess our model’s robustness. The entire dataset is initially divided into five equal segments or folds. Each fold is reserved as the test set and the remaining four folds are combined to form the training set.

**FIGURE 4:**
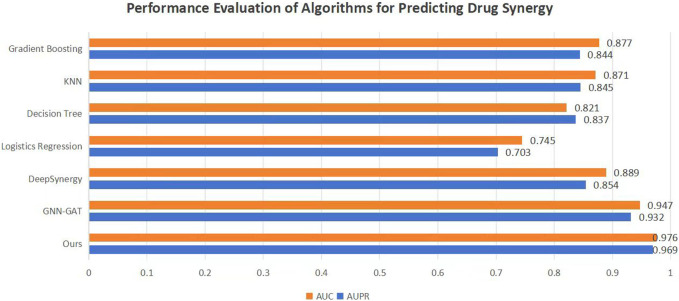
Performance evaluation of algorithms for predicting drug synergy. The bar chart compares the AUC and AUPR metrics across various algorithms used for predicting drug synergy. Each algorithm’s performance is illustrated by a pair of bars, one for AUC and the other for AUPR, highlighting their effectiveness in synergy prediction.

To validate the efficacy of the proposed DKPE-GraphSYN approach, we conducted comparisons with various benchmark models, including the well-known models DeepSynergy and GNN-GAT. The outcomes are displayed in Figure 4, with a focus on these typical benchmarks. Our model demonstrated the highest performance on both metrics, achieving an Area Under the Precision Recall Curve (AUPR) of 0.969 and an Area Under the Curve (AUC) of 0.976, representing an 11.5% improvement in AUPR over the benchmark model DeepSynergy while GNN-GAT also showed higher performance in AUC, it was lower than our model in AUPR. Among traditional machine learning algorithms, Gradient Boosting (Friedman, 2001) and K Nearest Neighbors (KNN) (Guo et al., 2003) performed similarly in AUC but were both lower than the DKPE-GraphSYN model. Based on these results, we can conclude that DKPE-GraphSYN performs better in predicting drug synergy effects compared to other algorithms. This conclusion is derived from aggregating the analyses of five separate experiments, affirming the dependability and uniformity of the findings. The comparison outcomes, not only validate the DKPE-GraphSYN model’s effectiveness in foreseeing drug synergistic effects but also highlight the noteworthy enhancement in performance due to the DKDE and position encoding channel cascading technique’s capability in managing intricate biological datasets.

The foundation of our prediction model lies in applying a network for regression analysis on the synergy scores of various drug combinations, leading to the calculation of an average synergy score. This calculated score is compared to a set threshold to ascertain if the drug interaction exhibits synergy or antagonism, thereby classifying the outcome. Essentially, our methodology transitions from regression to classification. Therefore, we utilize a combination of classification indicators, AUC and accuracy (ACC), along with the regression indicator Pearson Correlation Coefficient (PCC), to conduct a thorough mixed method evaluation. This approach ensures a more comprehensive understanding of our model’s efficacy. The following table presents the comparison results of our model against other Deep Learning (DL) techniques and four Machine Learning (ML) strategies on regression and classification tasks. As shown in Table 1, our model outperformed others under the AUC metric, followed by AudnnSynergy (Zhang et al., 2021) and DeepSynergy. Under the ACC metric, the accuracy of most methods was very close, around 0.92 or 0.93, showing similar in accuracy between DL and ML methods. Under the PCC metric, DKPE-GraphSYN achieved a value of 0.84, meaning that the predicted values of our model are highly consistent with the actual drug synergy scores, demonstrating a high degree of linear correlation. Based on these results, we infer that DL methods may be superior to ML methods in drug synergy prediction tasks, with DKPE-GraphSYN, particularly excelling in both AUC and PCC metrics. This superiority could be attributed to the DL methods’ capacity to manage complex and high dimensional data, along with the unique structural and algorithmic design of our model.

**TABLE 1:** Results of method comparison on the regression task and classification task.

Type	Method	AUC	ACC	PCC	RMSE
DL	Ours	0.97 ± 0.01	0.92 ± 0.04	0.83 ± 0.01	15.03 ± 1.21
DL	DeepSynergy	0.90 ± 0.03	0.92 ± 0.03	0.73 ± 0.04	15.91 ± 1.56
DL	AudnnSynergy	0.91 ± 0.02	0.93 ± 0.01	0.74 ± 0.03	15.46 ± 1.44
ML	Elastic Net (Zou and Hastie, 2005)	0.78 ± 0.04	0.92 ± 0.01	0.45 ± 0.02	20.41 ± 1.30
ML	SVM (Cortes and Vapnik, 1995)	0.88 ± 0.02	0.93 ± 0.01	0.63 ± 0.02	19.92 ± 1.28
ML	Random Forest (Li et al., 2017; Li et al., 2018)	0.87 ± 0.02	0.93 ± 0.01	0.64 ± 0.03	17.65 ± 1.13
ML	XGBoost (Celebi et al., 2019)	0.87 ± 0.02	0.93 ± 0.01	0.66 ± 0.02	17.16 ± 1.31

### 3.2 Experimental parameter settings

To maximize the performance of our model, extensive parameter tuning was conducted. Table 2 details main hyperparameter configurations and their impact on model performance. We set the batch size (batch_size) to 300 to balance computational efficiency and memory usage; We chose 100 epochs (num_epochs) to ensure comprehensive training and set the embedding layer dimension (emb_dim) at 100 to effectively capture features representations. Our model employs GATv2 as the graph neural network (gnn_type) and uses a 3 layer network (num_layer) to enhance the model’s learning capacity.

**TABLE 2:** Summary of training hyperparameters.

Hyperparameter name	Value	Description
batch_size	300	Number of graphs per training batch
num_epochs	100	Number of training epochs
emb_dim	100	Dimension of the embedding layer
gnn_type	GATv2	Type of Graph Neural Network
num_layer	3	Number of layers in the GNN

### 3.3 Ablation study

The foundation of our prediction model lies in applying a network for regression analysis on the synergy scores of various drug combinations, leading to the calculation of an average synergy score. This calculated score is then compared to a set threshold to determine if the drug interaction exhibits synergy or antagonism, thereby classifying the outcome. Essentially, our methodology transitions from regression to classification. Therefore, we utilize a blend of classification indicators, AUC, ACC and AUPR, alongside the regression indicator Root Mean Square Error (RMSE), PCC. This comprehensive approach ensures a more comprehensive understanding of our model’s efficacy.

In our study, we evaluated the roles of DKPE and GNN in drug combination synergy prediction models through a series of ablation experiments. Specifically, we examined four model variants: the complete model (both DKPE and GNN enabled), DKPE only, GNN only, and neither enabled. We assessed algorithm performance using the following metrics: AUPR, AUC, RMSE, PCC, and ACC. The results of the ablation study are shown in Table 3.

**TABLE 3:** Ablation study results for DKPE-GraphSYN.

DKPE	GNN	AUC	AUPR	PCC	ACC
√	√	0.976	0.969	0.843	0.923
√	×	0.912	0.893	0.653	0.830
×	√	0.880	0.847	0.609	0.809
×	×	0.860	0.821	0.540	0.770

**FIGURE 5:**
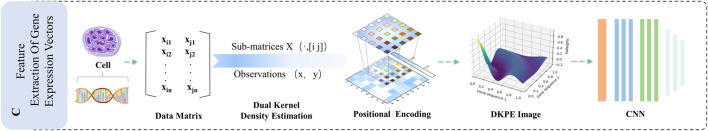
Illustration of the DKDE and PE Channel Concatenation Algorithm. This figure depicts the workflow from cell DNA to the creation of a DKPE image.

As depicted in Figure 5, the complete model demonstrated excellent predictive performance, achieving with an AUC of 0.976, an AUPR of 0.969, a PCC of 0.843, and an ACC of 0.923. These findings demonstrate that the effective integration of DKPE and GNN significantly enhance performance in synergy prediction. These results indicate that when the DKPE and GNN components are combined, the model is able to predict the synergistic effects of drug combinations with high accuracy.

When the GNN component was removed and only DKPE was used, we observed a decline across all performance metrics. The AUC decreased to 0.912, AUPR to 0.893, PCC fell to 0.653, and ACC dropped to 0.830. These results highlight the importance of GNN in capturing the complex interactions between drug combinations.

Conversely, retaining GNN while removing DKPE resulted in a decrease in AUC and AUPR to 0.880 and 0.847, respectively, a reduction in PCC to 0.609, with ACC at 0.809. This indicates that the DKPE component also plays a crucial role in enhancing the model’s predictive accuracy.

In the baseline model, where both KDE and GNN were not enabled, all performance metrics further decreased: AUC to 0.860, AUPR to 0.821, PCC dropped to 0.540, and ACC to 0.770. This reinforces the substantial impact of both KDE and GNN elements in enhancing the model’s overall predictive accuracy.

DKDE and PE are the key components of our algorithm, collectively referred to as DKPE. DKDE utilizes a Gaussian kernel function for weighted probability density calculation, smoothing gene expression data and reducing the impact of noise. Through DKDE, we vividly depict the interaction patterns and probability strength between genes. PE encodes the spatial distribution of gene expression features, aiding in understanding the relative positions of genes within a vector. This is crucial for interpreting gene interactions as gene effects are often influenced by their context and relative positions within the genome. PE enriches the data representation, providing a more nuanced analytical perspective. In summary, DKPE provides comprehensive and accurate analysis of gene expression, captures subtle features in gene expression data, and promotes a deep understanding of complex biological and chemical interactions.

Furthermore, we plotted Receiver Operating Characteristic (ROC) curves and Precision Recall (PR) curves to visually demonstrate the results of the ablation experiment.

### 3.4 Case study

**FIGURE 6:**
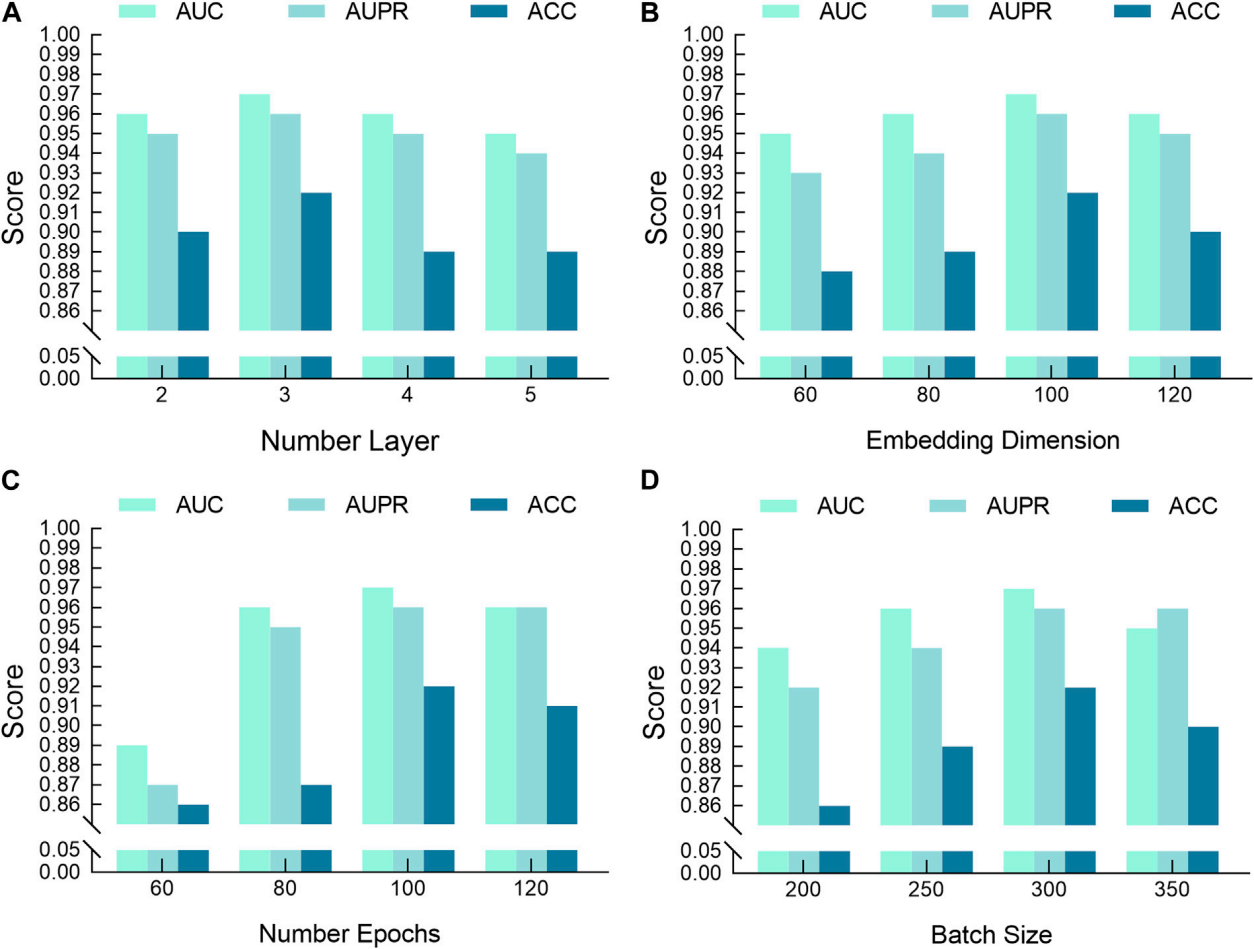
Variations in DKPE-GraphSYN Performance by Different Hyperparameters. **(A)** Scores by number layer; **(B)** Scores by embedding dimension; **(C)** Scores by number epochs; **(D)** Scores by batch size.

This section explores the impact of key network hyperparameters on the performance of our proposed DKPE-GraphSYN model. Figure 6 illustrates the variations in the AUC, AUPR, and ACC performance metrics under various parameter settings. Figure 6A shows changes in performance as the model’s number of layers increases from 2 to 5. The results indicate that performance improves with additional layers up to a threshold, beyond which performance gains diminish and may even decrease due to overfitting or unnecessary noise; Figure 6B depicts how varying the embedding dimension impacts performance. Increased dimensions initially enhance feature learning and model performance, but beyond a certain point, they may reduce performance due to overfitting to noise in the training data. Figure 6C highlights performance changes across different training epochs. Optimal results were observed at 100 epochs (AUC: 0.97, AUPR: 0.96, ACC: 0.92), with performance declining at 120 epochs. Therefore, 100 epochs were established as the ideal training duration. Figure 6D explores how different batch sizes affect the three key metrics. A batch size of 300 was optimal, balancing computational efficiency and training robustness while minimizing the risk of overfitting and promoting rapid model convergence.

**FIGURE 7:**
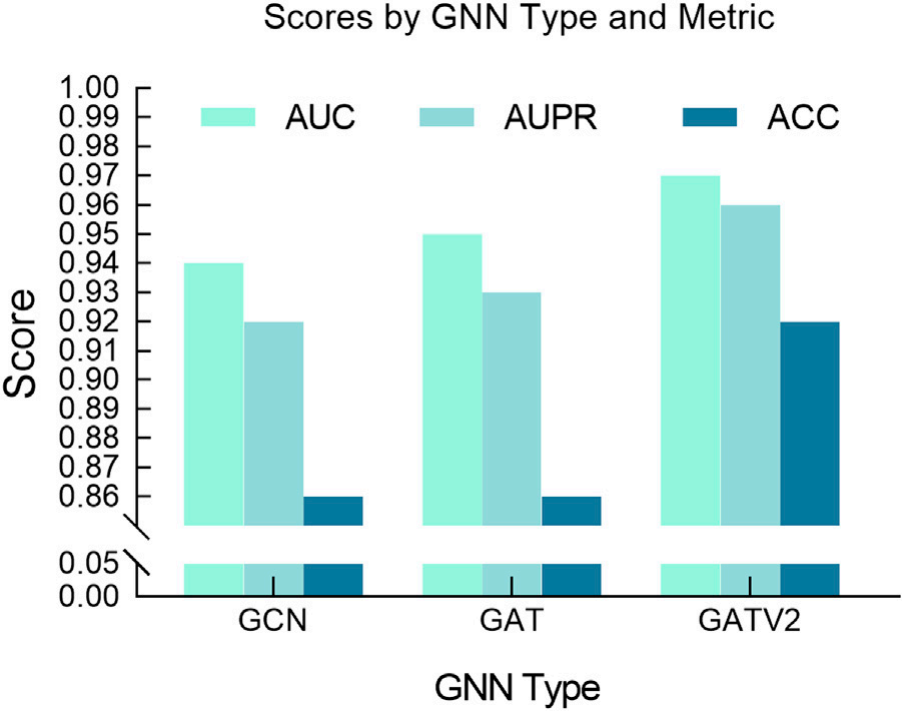
Comparative performance of different GNN models.

Additionally, we also studied the impact of different GNN architectures on model performance, focusing on the selection process between GATv2, GAT and GCN. GCN is a commonly used graph neural network model that propagates information by aggregating the features of neighbor nodes on each node. Compared with GCN, GATv2, an improved version of GCN, introduces dynamic attention coefficients, allowing the model to flexibly adjust the edge weights based on the current input features. As shown in Figure 7, it can be observed from our experimental results that GATv2 exhibits better performance and stability compared to GAT and GCN in processing drug graph data. By dynamically calculating attention coefficients, GATv2 can better capture the dependencies between drug molecules and adjust the weight of edges based on current input features, thereby adapting more effectively to various data scenarios during the learning process. Our experimental results show that GATv2 can provide higher accuracy and more stable performance when processing drug graph data, in contrast to GCN, which may be limited by fixed edge weights in the graph structure in some cases. Overall, the performance advantage of GATv2 in processing drug graph data is mainly reflected in its ability to dynamically adjust the weight of edges to better capture the relationship between drug molecules and maintain stable performance under different input features.

**FIGURE 8:**
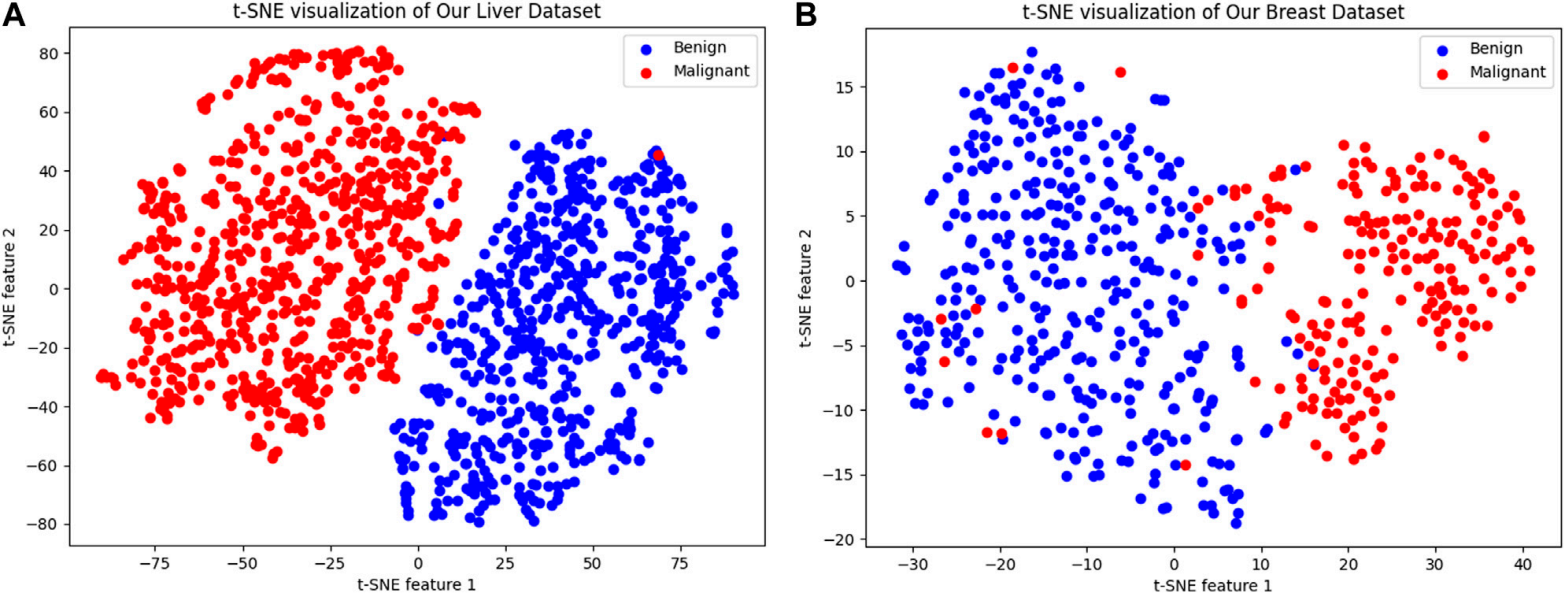
t-SNE Visualization of Cancer Cell Datasets. **(A)** The left panel presents a t-SNE visualization of the “Liver Dataset,” where each dot represents a sample, with malignant samples colored in red and benign samples in blue, forming two distinct clusters that demonstrate the model’s ability to differentiate between the two sample types in a two**-**dimensional space. **(B)** The right panel displays the t-SNE visualization for the “Breast Dataset,” similarly using red to denote malignant samples and blue for benign ones, clearly separating the two into discernible clusters. This visualization method highlights the model’s capability to identify and capture key features that distinguish between benign and malignant samples, which is critical for the subsequent calculation and prediction of synergy scores.

Figure 8 illustrates the outcomes of employing t-SNE (t-distributed Stochastic Neighbor Embedding) for visualizing various cancer cell datasets. t-SNE is a in machine learning technique that reduces the dimensionality of high dimensional data for easier visualization. In the depicted figures, each dot represents a sample, with the color indicating the sample’s classification. The figure on the left shows the t-SNE visualization for the “Liver Dataset,” while the figure on the right demonstrates it for the “Breast Dataset.” In both visualizations, malignant samples are marked in red, and benign samples are shown in blue.

We can clearly see that blue and red form two distinct clusters, indicating that our model can significantly differentiate between benign and malignant samples in a two-dimensional space. Despite the complexity of sample characteristics, our model captures the key features distinguishing the two types of samples, which is beneficial for subsequent calculation and prediction of synergy scores.

### 3.5 Evaluation on independent test set

To evaluate the generalization capabilities of our model, we conducted tests on a distinct, independent dataset: the NCI ALMANAC. Developed by the National Cancer Institute, this extensive screening resource is designed to identify anticancer drug combinations with enhanced therapeutic activity. Our independent testing involved 4,842 drug combinations derived from 100 drugs and 46 cell lines, amounting to a total of 215,946 entries.

Our model demonstrated exceptional performance in the independent tests, as evidenced by several key performance indicators. The AUC and the AUPR reached 0.962 and 0.957, respectively, indicating the model’s high accuracy in distinguishing drug synergistic effects. Additionally, the model achieved remarkable scores in ACC, F1 score, and recall, with values of 0.907, 0.909, and 0.925, respectively, further affirming its superior ability to accurately identify drug synergy.

In summary, these evaluation results highlight our model’s outstanding performance on new datasets and validate its robust generalization and interpretative power. This indicated that the model is not only effective on familiar data but also adapts well to and accurately recognizes entirely new and unseen data, demonstrating its potential for broad applicability in real world applications.

## 4 Discussion

The development and validation of the DKPE-GraphSYN model represent a significant advancement in the field of drug synergy effect prediction. This model’s innovative approach, which integrates DKDE and PE with graph neural networks, offers a nuanced method for capturing the complex interactions and structural features of drug molecules. The methodology distinguishes itself from existing models by providing a more detailed representation of gene expression data and drug molecular structures, addressing limitations highlighted in previous studies. The experimental results, demonstrate superior performance metrics with an AUPR of 0.969 and AUC of 0.976, compared to benchmark models. These findings validate the effectiveness of DKPE-GraphSYN in predicting drug combination synergy effects. These results highlight not only a testament to the model’s accuracy but also its reliability, marking a notable improvement over traditional and contemporary models like DeepSynergy and GNN-GAT. Such advancements underscore the potential of integrating sophisticated computational approaches with biological datasets to enhance predictive capabilities in drug discovery. The model’s success in accurately predicting drug synergy effects aligns with the growing recognition of the importance of computational models in personalized medicine and treatment efficacy enhancement. By leveraging the spatial distribution characteristics and weighted probability densities of gene expression alongside the structural regularity information of drugs, DKPE-GraphSYN provides a holistic view of the potential interactions between drug molecules. This comprehensive analysis is crucial for unraveling the complexities of drug interactions, offering a promising auxiliary method for clinical medication administration and new drug development.

Looking ahead, the implications of the DKPE-GraphSYN model extend beyond the current study. The model opens up new avenues for exploring combination therapies, particularly in cancer treatment, where the synergy between drugs can significantly impact therapeutic outcomes. The ability to accurately predict drug combinations that will exhibit synergistic effects can greatly aid in designing of more effective treatment regimens, potentially reducing side effects and improving patient quality of life. Furthermore, the insights gained from the DKPE-GraphSYN model can inform future research directions, such as the identifying of novel drug targets and the exploring uncharted pathways in drug interactions. Applying the model to a broader array of datasets and clinical scenarios could further validate its efficacy and adaptability, paving the way for its integration into clinical decision support systems. In conclusion, the DKPE-GraphSYN model not only exemplifies the power of advanced computational techniques in drug synergy prediction but also highlights the critical role of such models in the future of drug discovery and development. As we continue to refine and expand upon this model, its contribution to accelerating the discovery of drug combination therapies and enhancing patient treatment outcomes cannot be overstated. The continued exploration and development of such models are essential for advancing our understanding of drug interactions and their implications for personalized medicine.

## 5 Conclusion

The DKPE-GraphSYN model introduced in this study offers a pioneering approach to predicting the synergy effects of anticancer drugs. It analyzes the spatial distribution characteristics and weighted probability densities of gene expression vector data, while also exploring into the structured regularity and potential relationships among drug molecules specific to cancer therapy. By leveraging DKDE and PE, the DKPE-GraphSYN model effectively integrates of information at the channel level. The employment of graph neural networks for depicting drug molecules further enhances its capability to discern structural attributes of anticancer drugs. Experimental results show that our model substantially surpasses existing methods in forecasting the synergy effects of drug combinations, achieving an AUPR of 0.969 and an AUC of 0.976 on an extensive dataset of drug and cell line combinations for cancer treatment. This marks a substantial leap in the accuracy and reliability of cancer drug synergy prediction.

The DKPE-GraphSYN model not only enhances the precision in predicting anticancer drug synergy effects but also provides critical insights for clinical decision making and novel anticancer drug development. Moving forward, the continued refinement and application of the DKPE-GraphSYN model are expected to expedite the discovery of effective anticancer drug combinations, significantly improving treatment options for patients.

## Code availability statement

All methods are implemented in PyTorch and trained on an NVIDIA GeForce RTX 3090 GPU with 64GB of memory. Details such as hyperparameters and data preprocessing can be found in our implementation code at https://github.com/daxuede/DKPE-GraphSYN.

## Data Availability

The original contributions presented in the study are included in the article/supplementary material, further inquiries can be directed to the corresponding author.
